# Uterine leiomyoma with RAD51B::NUDT3 fusion: a report of 2 cases

**DOI:** 10.1007/s00428-023-03603-9

**Published:** 2023-07-19

**Authors:** Pavel Dundr, Alba Machado-Lopez, Aymara Mas, Zuzana Věcková, Michal Mára, Adéla Richtárová, Radoslav Matěj, Ivana Stružinská, Michaela Kendall Bártů, Kristýna Němejcová, Jiří Dvořák, Jan Hojný

**Affiliations:** 1 Department of Pathology, First Faculty of Medicine, Charles University and General University Hospital in Prague, Studničkova 2, 128 00 Prague 2, Czech Republic; 2 Carlos Simon Foundation, INCLIVA Health Research Institute, 46010 Valencia, Spain; 3 Department of Obstetrics and Gynecology, First Faculty of Medicine, Charles University and General University Hospital in Prague, Prague, Czech Republic; 4 Department of Pathology, Charles University, 3rd Faculty of Medicine, University Hospital Kralovske Vinohrady, Prague, Czech Republic; 5 Department of Pathology and Molecular Medicine, Third Faculty of Medicine, Charles University, Thomayer University Hospital, Prague, Czech Republic

**Keywords:** Uterine leiomyoma, Cellular leiomyoma, *RAD51B::NUDT3* fusion

## Abstract

Three main uterine leiomyoma molecular subtypes include tumors with *MED12* mutation, molecular aberrations leading to HMGA2 overexpression, and biallelic loss of FH. These aberrations are mutually exclusive and can be found in approximately 80–90% of uterine leiomyoma, in which they seem to be a driver event. Approximately 10% of uterine leiomyoma, however, does not belong to any of these categories. Uterine leiomyoma with HMGA2 overexpression is the most common subtype in cellular and second most common category of usual leiomyoma. In some of these tumors, rearrangement of *HMGA2* gene is present. The most common fusion partner of *HMGA2* gene is *RAD51B*. Limited data suggests that *RAD51B* fusions with other genes may be present in uterine leiomyoma. In our study, we described two cases of uterine leiomyoma with *RAD51B::NUDT3* fusion, which occur in one case of usual and one case of highly cellular leiomyoma. In both cases, no other driver molecular aberrations were found. The results of our study showed that *RAD51::NUDT3* fusion can occur in both usual and cellular leiomyoma. *RAD51B* may be a fusion partner of multiple genes other than *HMGA2* and *HMGA1.* In these cases, *RAD51B* fusion seems to be mutually exclusive with other driver aberrations defining molecular leiomyoma subtypes. *RAD51B::NUDT3* fusion should be added to the spectrum of fusions which may occur in uterine leiomyoma, which can be of value especially in cellular leiomyoma in the context of differential diagnosis against endometrial stromal tumors.

**Supplementary Information:**

The online version contains supplementary material available at 10.1007/s00428-023-03603-9.

## Introduction

Among uterine mesenchymal tumors, which represent a heterogeneous group of tumors arising from the smooth muscle and connective tissue of the uterus, uterine leiomyomas, also known as fibroids, are the most common and most important benign tumors with an estimated lifetime incidence of up to 70% [1].

Although current medical diagnosis of uterine mesenchymal tumors is based mainly on imaging and histological procedures, molecular tools are rapidly expanding and gaining relevance as a complement to conventional strategies in all clinical fields [2–5]. In this sense, understanding the molecular aberrations can be of diagnostic value, as they can help distinguish between different types of uterine mesenchymal tumors with similar clinical and pathological features. In some cases, this knowledge can also be of therapeutic value. To date, some of the reported targetable aberrations include, for example, *ALK*, *NTRK*, *ROS1,* and other tyrosine kinase receptor rearrangements. The three main uterine leiomyoma molecular subtypes include (i) tumors with *MED12* point mutations, (ii) tumors with biallelic loss of *FH,* and (iii) tumors with HMGA2 overexpression, commonly associated with chromosomal rearrangements (in HMGA1/HMGA2 or COL4A5/COL4A6), mainly resulting in the overexpression of these genes or reduced expression of CUX1 or CUL1 due to 7q deletions [6–11]. Interestingly, while the latter genetic alteration represents the second most common category of usual-type leiomyoma, it is even more commonly found in cellular leiomyoma, where almost all cases (over 90%) show HMGA2 overexpression [12–14]. In some of these tumors, rearrangement of *HMGA2* is present [15]. The most common fusion partner of *HMGA2* is *RAD51B* [16, 17]. However, the limited data suggests that fusions of *RAD51B* with other genes may also be present, and they seem to be mutually exclusive with other aberrations [18]. In our study, we described two cases of uterine leiomyoma with *RAD51B::NUDT3* fusion, which occur in one case of usual-type and one case of cellular leiomyoma. Our cases represent the first tumors in which *RAD51B::NUDT3* fusion has been found, but fusion of *RAD51B* with other genes than *HMGA2* and *NUDT3* can occur.

## Material and methods

The study included two cases of uterine leiomyoma with *RAD51B::NUDT3* fusion. One of them (case 1) was a routine diagnostic case from the Department of Pathology, First Medical Faculty and General University Hospital in Prague, in which the fusion was detected during the diagnostic work up. The second case (case 2) was uterine leiomyoma which was included in our previous study focused on genomic and transcriptomic profiling of uterine leiomyoma and uterine leiomyosarcoma [18].

### Immunohistochemical analysis

The immunohistochemical (IHC) analysis was performed using 4-μm-thick sections of formalin-fixed and paraffin-embedded (FFPE) tissue. The list of antibodies used, including their clones, manufacturers, dilution, and staining instruments, is summarized in Supplementary Table 1.

### Exome and transcriptomic next generation sequencing analysis

DNA and RNA were isolated and characterized as described before [19].

One microgram of total RNA (>200bp) from tumor and non-tumor tissue was used for the rRNA and globin mRNA depletion using NEBNext Globin & rRNA Depletion Kit (New England Biolabs). Transcriptome RNA-Seq libraries were constructed using KAPA RNA HyperPrep Kit according to the Roche KAPA HyperCap Workflow v3.2 with minor modifications (RNA fragmentation 65°C for 2 min; KAPA UMI adapters were used at a final concentration of 750 nM; total 13 PCR cycles using KAPA UDI Primer Mixes). Exome DNA libraries from tumor tissue only were prepared using KAPA HyperExome probes (Roche) by KAPA HyperCap Workflow v3.2 as described before [19].

Exome and transcriptome libraries were sequenced using NextSeq 500 (Illumina) and High Output Kit v2.5 (300 cycles), with target of 300 million reads per exome (reached average coverage was: 258×, case 1, and 358×, case 2), 60 million reads per tumor (reached reads output was 63.3 million, case 1, and 69.7 million, case 2), and 30 million reads per non-tumor transcriptome (reached reads output was 22.3 million, case 1, and 50.4 million, case 2).

Bioinformatic analysis of raw sequencing data, genomic variants annotation, tumor mutation burden (TMB) calculation, and fusion detection was processed as described before [19]. Tumor vs paired non-tumor tissue differential expression analysis was performed using the Differential Expression in Two Groups module in CLC Genomics Workbench v23.0.2 software (CLC GW; Qiagen). Only validated genes (according to the NCBI RefSeq) with a transcript per million (TPM) value above 40 were evaluated in gene expression analysis. Only significant differences ≥ 10-fold were reported.

## Results

### Morphological and immunohistochemical findings

**Fig. 1:**
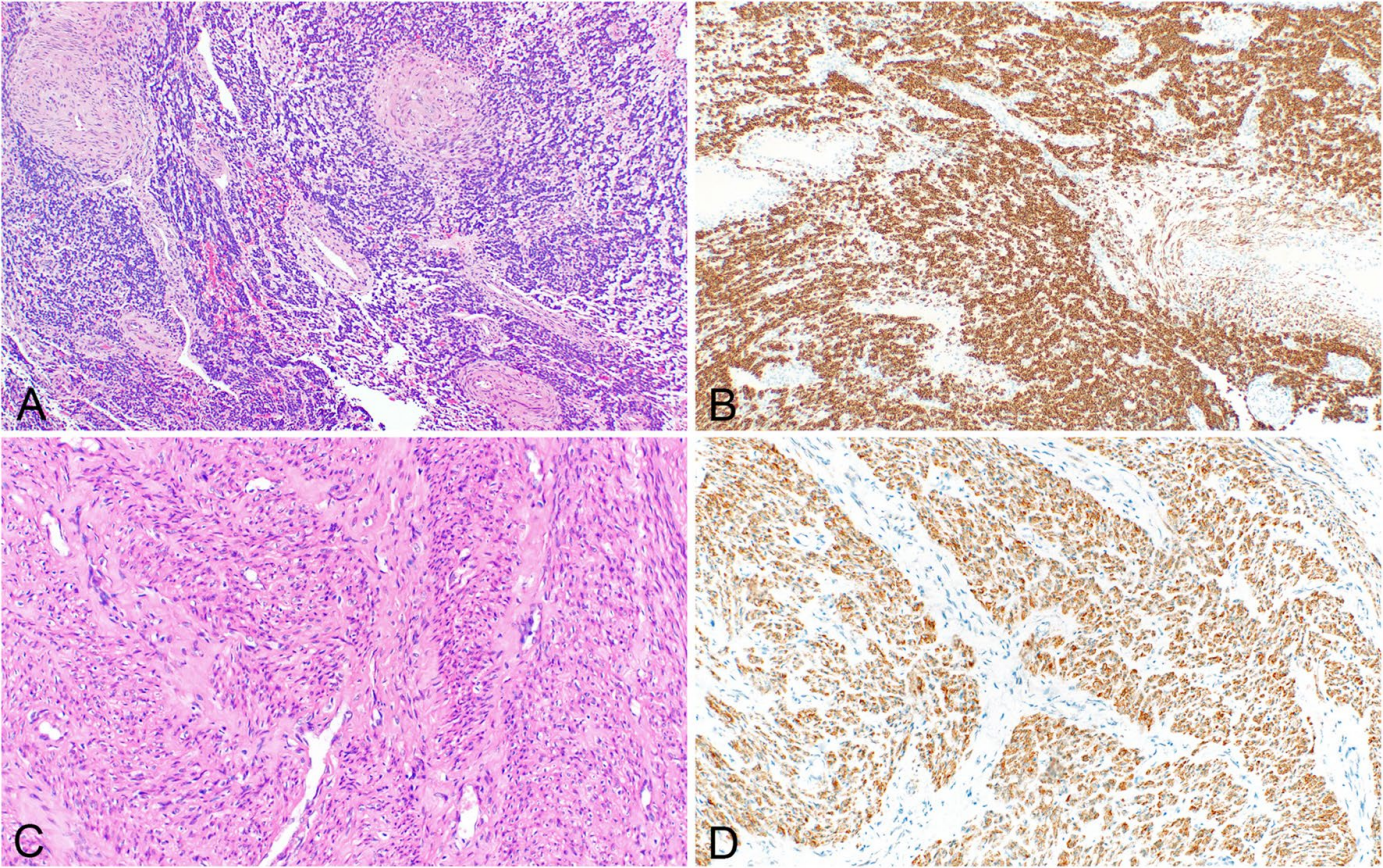
Case 1, cellular leiomyoma with a substantially increased cellularity (1**A**) (HE, 100×). Immunohistochemical positivity of tumor cells for desmin (1**B**) (200×). Case 2, usual type leiomyoma consisting of spindle cells with regular nuclei (1**C**) (HE, 200×). Immunohistochemical positivity of tumor cells for transgelin (1**D) **(200×)

The first case was a 39-year-old female referred to our institution for laparoscopic myomectomy due to persistent uterine bleeding. The ultrasonography before procedure showed nodular tumor mass 50 mm in diameter. The patient underwent laparoscopic myomectomy with in-bag morcellation. Macroscopically, the material submitted to biopsy examination consisted of multiple tumor fragment weighting 25 g. Microscopically, the tumor was highly cellular and consisted of spindle or oval tumor cell with regular nuclei and small amount of cytoplasm (Fig. 1A). The lesion showed irregular demarcation from myometrium. Immunohistochemically, the tumor cell showed positivity for desmin, smooth muscle actin and CD10 (Fig. 1B). PLAG1 showed weak positivity in most tumor cells. H-caldesmon and IFITM1 showed focal weak positivity. HMGA2, transgelin, NTRK, BCOR, S100 protein, and BCORL1 were negative. The diagnosis was highly cellular leiomyoma, but due to some equivocal features, molecular testing was performed to exclude possibility of tumor with endometrial stromal differentiation.

The second case was analyzed in our previous study [18]. The patient was a 45-year-old female who underwent laparotomic hysterectomy due to leiomyoma-related symptomatology and presented with an intrauterine mass 70 mm in diameter. Microscopically, the tumor had features of usual-type leiomyoma consisting of spindle cells without nuclear atypia and mitoses (Fig. 1C). Immunohistochemically, the tumor was positive for transgelin, desmin, and h-caldesmon (Fig. 1D). IFITM1, PLAG1, and HMGA2 were negative. No other clinical or pathological data are available for this case.

### Molecular findings

#### DNA sequencing

DNA exome sequencing did not reveal any likely pathogenic or pathogenic (class 4–5) variant in any cancer-related gene in both analyzed cases. Low somatic mutation levels were detected in both samples, 56 somatic variants in case 1 (TMB = 1.7 mut/Mb) and 66 somatic variants in case 2 (TMB = 1.9 mut/Mb), respectively.

#### RNA sequencing

**Fig. 2:**
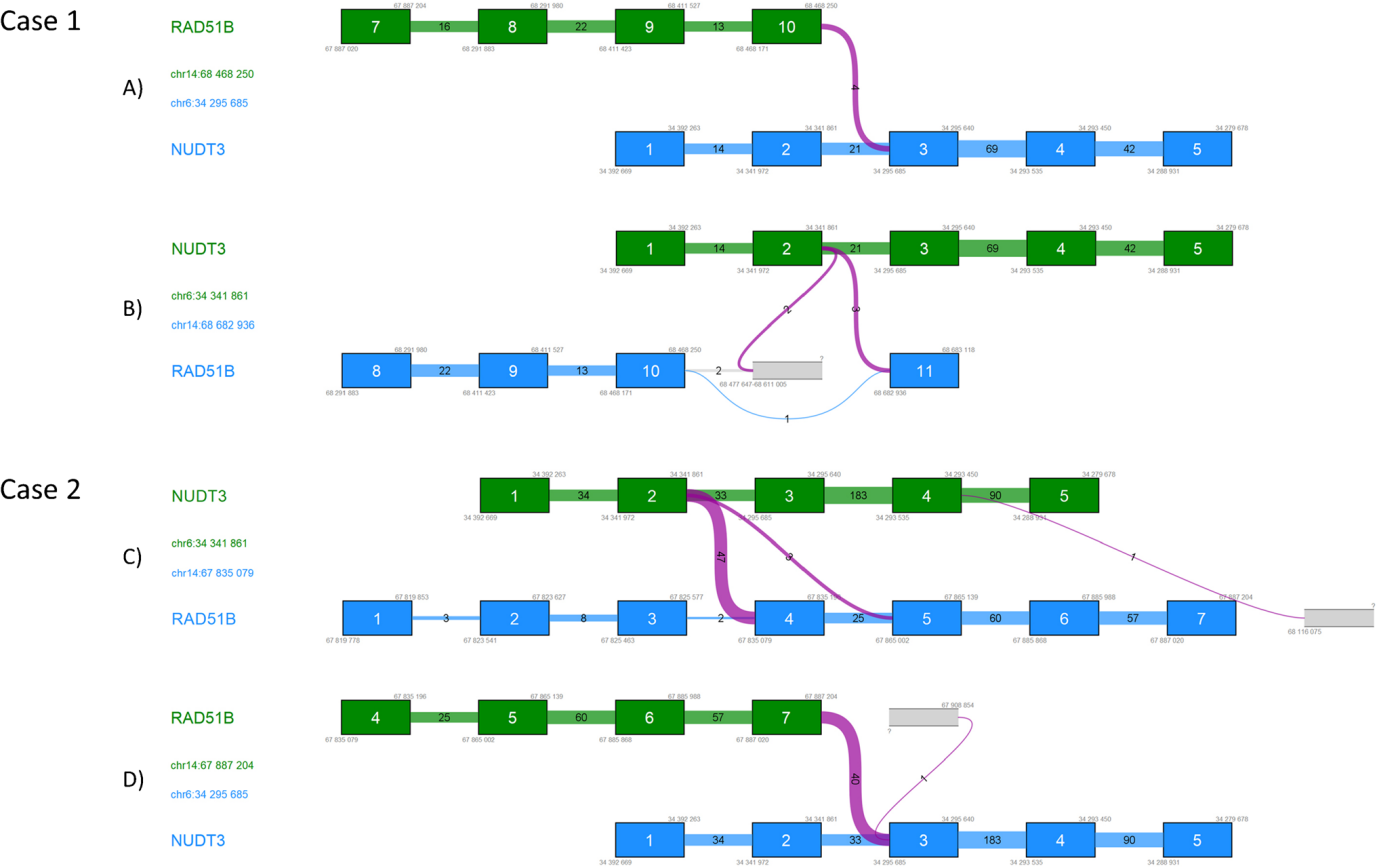
Detected *RAD51B* fusions. Diagrams of four detected *RAD51B* fusions (A–D) exported from CLC Genomics Workbench (Qiagen). Green, 5’-gene; blue, 3’-gene; blue/green boxes, numbered respective exons; blue/green lines, canonical exon-exon junctions with number of crossing reads; violet lines, fusion exon-exon junctions with a number of fusion crossing reads. Frameshift fusions (**A**) *RAD51B*(NM_133509.4):r.1_1113_*NUDT3*(NM_006703.4):r.518_9900 and (**B**) *NUDT3*(NM_006703.4):r.1_517_*RAD51B*(NM_001321818.1):r.1114_1295 in cellular leiomyoma (case 1). In-frame fusions (**C**) *NUDT3*(NM_006703.4:r.1_517_*RAD51B*(NM_133509.4):r.276_2659 and (**D**) *RAD51B*(NM_133509.4):r.1_833_*NUDT3*(NM_006703.4):r.518_9900 in usual leiomyoma (case 2)

Detected fusions are depicted in Fig. 2. In case 1, *RAD51B*::*NUDT3* fusion transcript was detected in 4 unique crossing reads, *RAD51B*(NM_133509.4):r.1_1113_*NUDT3*(NM_006703.4):r.518_9900, which connects exon 10 of *RAD51B* with exon 3 of *NUDT3*. Furthermore, *NUDT3*::*RAD51B* opposite fusion transcript was detected in 3 unique crossing reads, *NUDT3*(NM_006703.4):r.1_517_*RAD51B*(NM_001321818.1):r.1114_1295, which connects exon 2 of *NUDT3* with exon 11 of *RAD51B*. Both fusion events have disrupted open reading frame. In case 2, *NUDT3*::*RAD51B* fusion transcript was detected in 47 unique crossing reads, *NUDT3*(NM_006703.4:r.1_517_*RAD51B*(NM_133509.4):r.276_2659, which connects exon 2 of *NUDT3* with exon 4 of *RAD51B*. An opposite fusion event *RAD51B*(NM_133509.4):r.1_833_*NUDT3*(NM_006703.4):r.518_9900 which fused exon 7 of *RAD51B* and exon 3 of *NUDT3* was detected in 40 unique crossing reads. Both fusions maintain original open reading frame. In corresponding non-tumor counterparts, none of these fusion events was detected.

Although the reported fusions had different breakpoints inside *NUDT3* and *RAD51B* genes, due to the detection of opposite *RAD51::NUDT3* fusion with similar number of fusion crossing reads, we can assume that detected translocation is balanced and reciprocal between chr14 (q24.1) and chr6 (p21.31) in both tumor cases.

### Expression mRNA analysis

Significant changes in tumor expression (≥ 10-fold) with the same trend in both cases are summarized in Table 1. Overview of all significant ≥ 10-fold up- and downregulated genes and list of all significant ≥ 2-fold changes in tumor expression are in Supplementary Table 2.

**Table 1:** Significantly upregulated and downregulated genes in uterine leiomyomas

Gene	Case 1		Case 2		RefSeq Gene ID	RNA type
	Max TPM	Fold change	Max TPM	Fold change		
*Upregulation*						
*PLAG1*	114.53	53.96	41.65	44.38	5324	Protein coding
*CD24*	395.56	15.99	598.15	58.36	100133941	Protein coding
*SNORA48*	264.38	14.68	231.18	19.16	652965	snoRNA
*CAPN6*	93.19	14.54	983.94	46.86	827	Protein coding
*HMGA1*	254.61	13.14	609.06	50.86	3159	Protein coding
*SNORD10*	203.64	12.41	194.34	11.99	652966	snoRNA
*Downregulation*						
*DKK3*	44.12	−16.99	119.64	−12.72	27122	Protein coding
STK26*	48.41	−36.78	233.56	−12.75	51765	Protein coding

Only significant ≥ 10-fold RNA expression changes with same trend in cellular leiomyoma (case 1) and usual leiomyoma (case 2) are listed. Max TPM represents maximum expression value in tumor or non-tumor tissue. Fold change was calculated by differential expression analysis comparing tumor sample and non-tumor counterpart. *snoRNA*, small nucleolar RNA; *TPM*, transcripts per million; *STK26 (also known as *MST4*)

Significant change of expression, reaching 10- to 58-fold change between uterine lesion compared to non-tumor counterpart, was observed in *CAPN6*, *CD24*, *HMGA1*, *PLAG1*, *SNORA48*, and *SNORD10* (upregulation) and *DKK3* and *STK26* (downregulation).

## Discussion

The etiopathogenesis of uterine leiomyoma has been studied from several aspects including their molecular features. It has been shown that three main uterine leiomyoma molecular subtypes exist, including tumors with *MED12* mutation, molecular aberrations leading to HMGA2 overexpression, and biallelic loss of *FH* [8, 10, 11, 20–23]. These mutually exclusive aberrations seem to be a driver event and can be detected in approximately 80–90% of uterine leiomyomas. However, the frequency of molecular aberration occurring in uterine leiomyoma differs between leiomyoma subtypes. Most usual uterine leiomyomas (40–75%) are characterized by *MED12* mutation, followed by 10–25% with HMGA2 overexpression [6, 20]. Specifically, in cellular leiomyoma, the most common is HMGA2 alteration (35% of cases), followed by chromosome 1p deletion (up to 25% of cases) and *MED12* mutation (5–16% of cases) [24, 25]. In our previous study on cellular leiomyoma, deletion of chromosome 1p was mutually exclusive with other driver alterations [13]. However, the specific gene affected by this deletion is currently unknown [20]. FH alterations are mostly restricted to FH-deficient leiomyoma and leiomyoma with bizarre nuclei [22, 26, 27]. The incidence of *FH* alterations in usual and cellular leiomyoma is very rare, in the range 0–2.5% and 0–4%, respectively [10, 14].

Approximately 10% of uterine leiomyomas, however, does not belong to above-described categories. From these tumors, 38% in one study showed overexpression of HMGA1 [28]. Another study focused on 111 tumors, which were classified as negative for driver alteration based on Sanger sequencing and immunohistochemistry [17]. Forty-three of these tumors (39%) showed features typical for HMGA2-altered tumors including PLAG1 overexpression and 16 of them (14%) chromosomal rearrangements of *HMGA2* (despite not having overexpression of HMGA2 by IHC), *HMGA1*, or *PLAG1*. HMGA1 and PLAG1 aberrations are not mutually exclusive with other alteration and can co-occur with *MED12* mutation. Based on this, they have been suggested to be a secondary event related to tumor progression. Nevertheless, aberrations of both genes can occur also as an isolated finding and can be a driver event in uterine leiomyoma [17]. Other rare molecular driver aberrations occurring in uterine leiomyoma included somatic mutations in genes encoding six members of SRCAP histone-loading complex leading to H2A.Z loading defect [28]. It has been proved that patients with germinal mutation in the SRCAP members *YEATS4* and *ZNHIT1* predispose to uterine leiomyoma [28].

One recent study suggested the leading role of HMGA2 aberrations in uterine leiomyoma tumorigenesis, which is overexpressed even in leiomyomas with *MED12* mutation [29]. However, the data in our previous study showed in three *MED12* mutated cases neither HMGA2 overexpression on IHC level nor increased *HMGA2* mRNA [13]. Some cases with HMGA2 overexpression are associated with *HMGA2* translocations or aberrant splicing, but in most studies, simultaneous analysis of IHC expression and molecular aberrations was not performed and the exact incidence of cases showing *HMGA2* rearrangement is not clear [30, 31]. The most common fusion partner of *HMGA2* is *RAD51B* [16, 17, 32]. Other mechanisms potentially involved in HMGA2 overexpression are hypomethylation and regulation by the microRNA Let-7 family [31, 33, 34]. While it has been suggested that alteration of HMGA2 is considered to be the initial step leading to significant upregulation of PLAG1, the role of RAD51B should not be overlooked, as it is also important in uterine leiomyoma development [20]. In one study including 8 cases with *HMGA2* rearrangement, 4 showed fusions with *RAD51B*, two with *PTGER*3, and two were rearranged without candidate partner gene [17]. Furthermore, other 4 cases in this study showed *HMGA1* fusions, two with *RAD51B*, one showing complex rearrangement involving *TRAF3IP2* and *PRDM1*, and one with *PBX1*. In our previous study on cellular leiomyoma, 33% (5/15) of tumors with HMGA2 overexpression showed *HMGA2* rearrangement [13]. The fusion partners include *C9orf92*, *PBX1*, and *RAD51B*. In two cases, no fusion partner genes were found. In both cases, the rearrangement was within non-coding areas of chromosome 5. Another study comparing uterine leiomyoma and leiomyosarcoma found that a small percentage (3 out of 56) of leiomyoma cases showed a *RAD51B* fusion (with *HMGA2*, *NCOR2*, and *NUDT3)*, and one of these cases with *RAD51B::NUDT3* fusion is reported here in detail [18]. Interestingly, disruptions in *NUDT3* have been shown to enhance cell migration in tumorigenic processes [35].

The expression of *RAD51B* and *NUDT3* was detected on similar levels, which supports the hypothesis of balanced and reciprocal translocation event leading to these fusions.

While both cases showed 5-fold upregulation of *RAD51B* expression when compared to the matched healthy myometrium, there was no change in *NUDT3* expression in case 1, with case 2 showing only 2-fold increased expression compared to the matched myometrium. Our findings support the previously published data suggesting that these fusions lead to the loss of physiological functions of RAD51B and NUDT3, resulting in a tumorigenic process. Concerning other RNA expression findings, transcriptional differences among leiomyomas harboring different genetic drivers have been described. Significant upregulation of *PLAG1* was described in HMGA2 subtype of leiomyoma [20]. Overexpression of this gene can be also associated with upregulation of insulin-like growth factor-2 (*IGF2*) [36]. Moreover, overexpression of *HMGA1* and/or *HMGA2* is in leiomyomas common finding [20]. In our study, we have found upregulation or downregulation of several genes. In concordance with literary data, upregulation of *HMGA1* and *PLAG1* mRNA was detected in both cases. The *HMGA2* mRNA expression in tumor and non-tumor tissue was below the level of reliable evaluation of expression pattern, which is in line with IHC negative results. Immunohistochemically detected PLAG1 protein expression showed weak positivity in case 1 and negativity in case 2. The *IGF2* mRNA upregulation (≥ 10-fold) was observed only in usual leiomyoma. Furthermore, highly upregulated mRNA of cell surface marker *CD24* was detected in our cases which correlates with previous findings of enriched CD24hi cells in leiomyoma. Another upregulated gene *CAPN6* was detected also in both cases. Its upregulation has been previously described in uterine leiomyoma and was shown to be involved in proliferation and apoptosis while being mediated through the Rac1/PAK1 signaling pathway [37]*. SNORA48* and *SNORD10* (coding for small nucleolar RNAs) have not yet been described in leiomyomas, however were upregulated in both our cases. Some snoRNAs exhibit differential expression patterns in a variety of human cancers [38]. In one study, *SFRP1* was significantly upregulated in leiomyomas relative to normal adjacent myometrium while other Wnt inhibitors such as *APC*, *DKK1*, and *DKK3* were significantly downregulated [39]. We observed downregulation of *DKK3* in both samples. Expression of *SFRP1* was downregulated in cellular leiomyoma and upregulated in usual leiomyoma. The expression of *APC* and *DKK1* was low and not reliable for evaluation of expression pattern. Furthermore, *STK26 (*previously known as *MST4)* downregulation was observed in both samples. This finding correlates with *MST4* downregulation in leiomyomas relative to normal myometrium reported previously [40].

The knowledge about molecular features of uterine leiomyoma can be of practical value in differential diagnosis especially in tumors with some unusual morphological features, such as cellular leiomyomas. In some of these tumors, especially so-called highly cellular leiomyomas, the distinction from tumors with endometrial stromal differentiation, including low grade endometrial stromal sarcoma (LG-ESS), may be problematic. Most of these tumors can be distinguished based on combination of morphological and immunohistochemical features. However, rare tumors can have overlapping features between cellular leiomyoma and LG-ESS and in these tumors, molecular testing may be helpful. However, the knowledge of molecular aberrations occurring in endometrial stromal tumors is rapidly evolving and the spectrum of aberration is broadening. These aberrations do not occur in uterine leiomyoma. However, with increasing knowledge about molecular aberrations occurring in mesenchymal uterine tumors, new aberrations were described, which can occur in both cellular leiomyoma and endometrial stromal tumors. For example, tumors with *KAT6B::KANSL1* and *KAT6A::KANSL1* fusion resembling LG-ESS some of them with sex cord-like features have been described recently [41]. These tumors have potential to aggressive behavior, even though most of them were characterized by well-defined borders. However, the fusions detected in these tumors have been described in 1 case of uterine leiomyoma and 1 case of uterine leiomyosarcoma [42, 43].

In conclusion, our study showed that *RAD51::NUDT3* fusion can occur in both usual and cellular leiomyoma. *RAD51B* may be a fusion partner of *HMGA2* and *HMGA1* but can occur in fusion with other genes including *NUDT3* and seems to be a potential driver event in these tumors mutually exclusive with other driver aberrations defining molecular leiomyoma subtypes. Nevertheless, more data is needed to confirm the possibility of *RAD51B* altered uterine leiomyoma as a distinct molecular subtype. From practical point of view, we should add the *RAD51B::NUDT3* fusion into the spectrum of fusions which can occur in leiomyocellular tumors, but has never been described in tumors of other histogenesis including inflammatory myofibroblastic tumor, endometrial stromal tumors, and tumors with kinase fusions such as *NTRK*, *RET*, and *ROS1*. Based on this, this fusion seems to be specific for tumors with leiomyocellular differentiation. This can be important for differential diagnosis between cellular leiomyoma and LG-ESS, but also for the differential diagnosis of tumors of other histogenesis, which can be in some cases with equivocal features complicated on morphological and immunohistochemical level only.

### Supplementary information

ESM 1

ESM 2

## Data Availability

NGS raw data available on request.
